# TiVZrNb Multi-Principal-Element Alloy: Synthesis Optimization, Structural, and Hydrogen Sorption Properties

**DOI:** 10.3390/molecules24152799

**Published:** 2019-07-31

**Authors:** Jorge Montero, Claudia Zlotea, Gustav Ek, Jean-Claude Crivello, Lætitia Laversenne, Martin Sahlberg

**Affiliations:** 1 Université Paris Est, ICMPE (UMR 7182), CNRS, UPEC, F-94320 Thiais, France; 2 Department of Chemistry, Uppsala University, Box 523, SE-75120 Uppsala, Sweden; 3 University Grenoble Alpes, CNRS, Grenoble INP, Institut Néel, 38000 Grenoble, France

**Keywords:** multi-principal element alloys, hydrogen absorption, phase transformation, neutron diffraction, thermo-desorption spectroscopy, SQS-DFT

## Abstract

While the overwhelming number of papers on multi-principal-element alloys (MPEAs) focus on the mechanical and microstructural properties, there has been growing interest in these alloys as solid-state hydrogen stores. We report here the synthesis optimization, the physicochemical and the hydrogen sorption properties of Ti_0.325_V_0.275_Zr_0.125_Nb_0.275_. This alloy was prepared by two methods, high temperature arc melting and ball milling under Ar, and crystallizes into a single-phase *bcc* structure. This MPEA shows a single transition from the initial *bcc* phase to a final *bct* dihydride and a maximum uptake of 1.7 H/*M* (2.5 wt%). Interestingly, the *bct* dihydride phase can be directly obtained by reactive ball milling under hydrogen pressure. The hydrogen desorption properties of the hydrides obtained by hydrogenation of the alloy prepared by arc melting or ball milling and by reactive ball milling have been compared. The best hydrogen sorption properties are shown by the material prepared by reactive ball milling. Despite a fading of the capacity for the first cycles, the reversible capacity of the latter material stabilizes around 2 wt%. To complement the experimental approach, a theoretical investigation combining a random distribution technique and first principle calculation was done to estimate the stability of the hydride.

## 1. Introduction

A very promising alloying strategy has emerged in the last decade based on the original concept of multi-principal-element alloys (MPEAs), where the alloy has significant atom fractions of several elements [1]. In contrast to classical alloy design that focuses on the optimization of compositions mainly located in the corners of phase diagrams (typically by adding small amounts of additional elements into a main phase), this new strategy emphasizes compositions in the center part of phase diagrams. Four or more principal elements are commonly alloyed with equal concentrations, but this is not compulsory. Among MPEAs, alloys with at least five principal elements with atomic concentrations between 5 and 35% are called high entropy alloys (HEAs) [2]. 

The alloying of many elements with high concentration and different atomic sizes may develop important lattice strain distortions. Large lattice distortion is particularly interesting for hydrogen storage since the creation of large interstitial sites might be beneficial for the insertion of significant amount of hydrogen. A quantitative parameter to describe the strained or distorted crystal lattice due to the mixing of many metals with different atomic radii is the lattice distortion or atomic size difference, *δ*, as defined in reference [2]. Empirical studies have shown that complete solid solutions are formed for *δ* < 6.6%. Distortions higher than this critical value may lead to the formation of multiphased alloys with intermetallic precipitations or even amorphous phases for δ > 8 [2].

These multi-principal-element alloys have received significant research interest in the last decades due to their excellent mechanical properties and potential industrial applications [3,4]. However, their functional properties such as, hydrogen sorption properties and/or resistance to hydrogen embrittlement, are only scarcely investigated [5]. Among the large variety of MPEAs compositions already reported, we are interested in *bcc* alloys based on refractory elements since these individual metals can absorb large hydrogen quantities forming hydride phases with maximum content of H/*M* = 2. Few reports have studied the hydrogen absorption in MPEAs based on refractory elements [6,7,8,9,10,11,12,13]. The first report on these materials explored the hydrogen storage properties of TiVZrNbMo synthesized by laser engineered net shaping [6]. This alloy showed a two-phase structure: a *bcc* solid solution and an orthorhombic NbTi_4_ intermetallic compound. The maximum hydrogen storage capacities were 2.3 wt.% just after synthesis and 1.8 wt.% after additional annealing. However, the hydrogen absorption induces phase segregation and formation of three distinct phases that hinder the reversibility of hydrogen absorption/desorption. Later, we reported a novel *bcc* alloy, TiVZrNbHf, that possesses improved hydrogen storage performances [7]. Interestingly, this alloy has a high storage capacity of 2.5 H/*M* (2.7 wt.%), which is larger than conventional transition metal hydrides (H/*M* = 2.0). X-ray and neutron diffraction experiments have pointed out that the initial *bcc* alloy undergoes a single step reaction with hydrogen thus forming a *bct* hydride phase with hydrogen occupying the tetrahedral sites [12]. Recently, the hydrogen absorption properties of the TiZrNbMoHf *bcc* alloy for solar thermal energy storage have been investigated [8]. This alloy can store reversibly around 1.2 wt.% (H/*M* = 1.2) hydrogen forming a hydride with *fcc* lattice. We have recently studied the TiZrNbHfTa *bcc* alloy with reversible hydrogen absorption/desorption properties [9]. This alloy undergoes a two-stage hydrogen absorption reaction to a *fcc* dihydride phase with an intermediate tetragonal monohydride similar to hydrogen interaction with classical *bcc* alloys. Very recently, a series of TiVZr*_z_*NbTa*_1-z_* and TiVZr*_1+z_*Nb alloys have been explored for hydrogen storage [10]. All alloys are single-phase with *bcc* structure and absorb hydrogen forming *fcc* hydrides with a maximum capacity close to, but never exceeding 2 H/*M*. However, the desorption from the Zr-rich hydrides (Zr > 12.5 at%) induces a phase segregation into two *bcc* structures. Thus, this study proves that Zr-rich MPEAs have poor reversibility towards hydrogen absorption/desorption. Another very recent study reported the structure and hydrogen sorption properties of a series of quaternary and quinary refractory MPEAs TiVNb*M* (*M* = Cr, Zr, Mo, Hf, Ta), TiZrNbHf, TiVCrNbMo, and TiVCrNbTa [13]. The alloys possess *bcc* structures and form *fcc* hydrides with H/*M* close to 2. This study suggests the valence-electron concentration (VEC) plays an important role in the destabilization of hydrides: the onset temperature for hydrogen desorption from the *fcc* hydrides decreases linearly with this parameter.

We report presently the synthesis optimization, the physicochemical and the hydrogen sorption properties of the refractory Ti_0.325_V_0.275_Zr_0.125_Nb_0.275_ MPEA with low Zr content. Along with classical high temperature melting technique for the synthesis of alloy, mechanochemical route under inert or reactive atmospheres has been employed to prepare the pristine alloy and the related hydride phase, respectively. The use of different synthetic techniques allowed us to optimize the microstructure of the alloy/hydride and to propose the material with the best performances. The capacity shows relatively good stability to around 2 wt.% for the material prepared by reactive ball milling after ten cycles, although a drop of 26% occurs during the initial cycles. Finally, X-rays and neutron diffraction helped identify the crystalline structure and the hydrogen occupation of the interstitial sites, respectively. 

In the past, several tentative attempts to describe MPEA by theoretical description have been performed but are only limited to metallic solid solutions [14,15,16]. In the present work, the hydride formation starting for the equimolar TiVZrNb composition has been studied by coupling several methodologies, as the special quasirandom structure (SQS) and the density functional theory (DFT).

## 2. Results

### 2.1. Synthesis of the TiVZrNb Alloy and Related Hydride

The nonequimolar Ti_0.325_V_0.275_Zr_0.125_Nb_0.275_ MPEA was synthesized by different techniques: high-temperature arc melting (HT-AM) and high energy ball milling under inert gas (BM). The chemical composition differs from the equimolar one and was defined to minimize the Zr content while still having a large lattice distortion, δ = 6%. Reactive ball milling (RBM) was used to synthesize the corresponding hydride phase under high pressure of H_2_. The availability of different synthetic routes enables us to study alloys/hydrides with different microstructure (different grain sizes, morphologies and surface area).

The HT-AM is the most common method to form refractory MPEAs with homogenous chemical composition, as demonstrated earlier [7,10,16]. The alloy is initially in the form of an ingot that was further used as a very coarse powder. 

The formation of the alloy by BM was checked at different process time to follow the phase formation during ball milling under Ar (Figure S1). Several phases are present after 15 min of ball milling which include some of the elemental metals and unknown contributions. However, the intensity of diffraction peaks from individual metals steadily decreases after 30 and 45 min and completely vanishes after 60 min, thus promoting the formation of a single phase. Further ball milling up to 150 min induces partial amorphization of the alloy, as proven by the decrease of the peaks’ intensities together with their broadening and the enlarged background contribution. Thus, 60 min of high energy ball milling under Ar is enough to prepare a single-phase MPEA.

**Figure 1:**
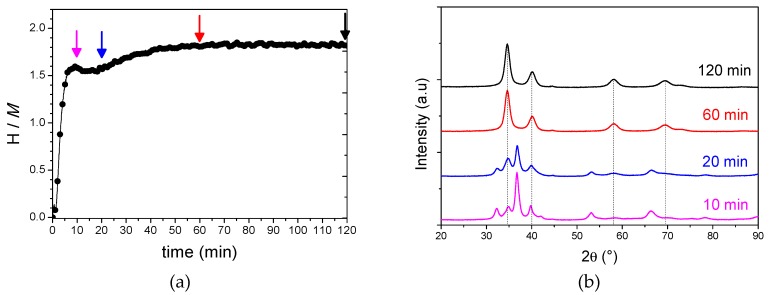
The hydrogen uptake profile during reactive ball milling of Ti_0.325_V_0.275_Zr_0.125_Nb_0.275_ under an initial pressure of 70 bar of H_2_ (**a**) and the XRD patterns after 10, 20, 60, and 120 min of milling process (**b**) (corresponding to the arrows in the left figure). The peaks originating from the final phase are indicated as dotted lines.

The hydrogen uptake (H/*M*) during RBM was determined from the pressure and temperature profiles recorded during the process and calculated as described earlier [17]. Figure 1a shows the hydrogen uptake profile with reactive ball milling time. A large hydrogen uptake up to around 1.6 H/*M* occurs in the first 5 min followed by a small plateau between 10 and 20 min and a second smooth increase that reaches a maximum of 1.8 H/*M* at 60 min. The uptake stabilizes with further ball milling time up to 120 min. In order to follow the phase evolution during RBM we have performed a structural investigation by XRD after 10, 20, 60, and 120 min (Figure 1b). 

After 10 min of RBM, just after the large hydrogen uptake, a mixture of elemental hydrides and unknown phases is present. The same peaks are also noticed after 20 min of RBM but the contribution from individual hydrides is reduced. A single-phase hydride is obtained only after 60 min of RBM and further ball milling does not change the structure nor the crystallinity of the phase. This finding highlights the complex reactions that take place during the RBM process. For the duration of the initial 5 min, the elemental powders are hydrogenated by a solid–gas reaction concomitant with a solid–solid reaction forming unknown phases, as attested by the XRD patterns and the large initial uptake up to 1.5 H/*M*. In the period from 10 to 20 min, only a solid–solid reaction seems to take place (plateau in Figure 1a), accompanied by a minor redistribution of phases: the diffraction peak at around 35°, corresponding to the main peak of the final phase, reinforces at the expenses of the other peaks. This solid–solid reaction continues with further ball milling together with a solid–gas reaction and slower kinetics, as the hydrogen uptake slightly increases from 1.5 to 1.8 H/*M* in the time period from 20 to 60 min. After 60 min of RBM, a single-phase hydride is obtained with the final composition Ti_0.325_V_0.275_Zr_0.125_Nb_0.275_H_1.8_. 

We have tested the efficiency of reactive mechanical alloying on the hydride formation (Figure S2). The speed rotation was decreased from initial 800 to 400 rpm while all other conditions were kept similar (70 bar H_2_, total sample mass = 5 g, ball-to-powder mass ratio 60:1, time = 120 min). The hydrogen uptake profile is different than the previous one and shows a continuous increase up to 2 H/*M* during the initial 40 min, followed by stabilization for further ball milling. Under these conditions, the final material is a mixture of different elemental hydrides, unknown phases and the final hydride. The XRD pattern resembles to the one at 800 rpm recorded after 20 min. Therefore, high energy reactive ball milling is essential to provide enough energy to complete the solid–solid reaction and to form a single-phase hydride. 

### 2.2. Crystalline Structure of the TiVZrNb Alloy and Related Hydride

**Figure 2:**
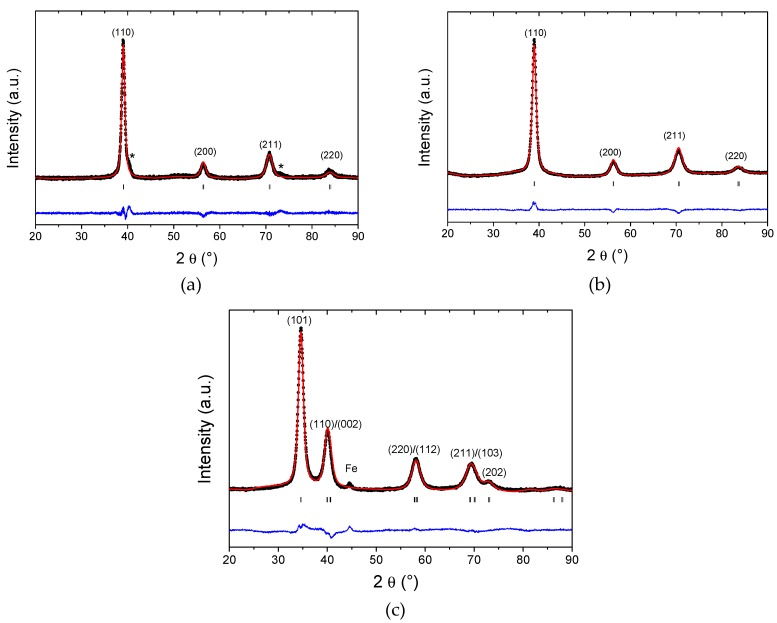
XRD patterns of the Ti_0.325_V_0.275_Zr_0.125_Nb_0.275_ alloy prepared by high-temperature arc melting(**a**), high energy ball milling under Ar (**b**), and the hydride phase Ti_0.325_V_0.275_Zr_0.125_Nb_0.275_H_1.8_ synthesized by reactive ball milling under H_2_ (**c**). Rietveld refinements results are also shown (calculated pattern in red and the difference curve in blue) together with the Miller indices of the corresponding phases.

After the optimization of the synthesis of MPEA and related hydride, the crystalline structure of all materials was investigated by XRD on powder samples. Figure 2 shows the XRD patterns of the pristine alloy prepared by HT-AM (a), by BM under Ar for 60 min (b) and by RBM under H_2_ for 120 min (c) together with the corresponding Rietveld refinements. Results of the structural refinements are listed in Table 1.

The HT-AM sample is almost single-phase with a very small impurity marked with stars in Figure 2a. This sample adopts a *bcc* structure ($Im\bar{3}m$) with the lattice parameter *a* = 3.261(1) Å (Table 1). The BM product is a single-phase material (Figure 2b) with the same structure and a slightly larger lattice parameter *a* = 3.270(1) Å (Table 1). The RBM hydride sample contains along with the main phase a small Fe impurity due to the preparation process (Figure 2c). The structural analysis of the hydride phase obtained by RBM can be carried out using either a *bct* or a *fcc* lattice. However, the refinement confidence factors are better for the *bct* lattice (χ^2^ = 6.4) than the *fcc* one (χ^2^ = 11.4) (Figure S3 and associated text). Thus, the *bct* lattice was preferred for RBM sample with the parameters *a* = 3.194(1) Å and *c* = 4.448(1) Å (Table 1). Moreover, the *bct* lattice can be understood as a slightly disordered *fcc* with a 5% expansion along the *a* axis and a 10% contraction along the *c* axis, as estimated from the lattice parameters (Figure S3 and related paragraph). In summary, the *bct* lattice is preferred for the hydride phase, irrespective of the type of synthesis of the alloys/hydrides.

### 2.3. Hydrogen Sorption Properties of the TiVZrNb Alloy Prepared by BM

**Figure 3:**
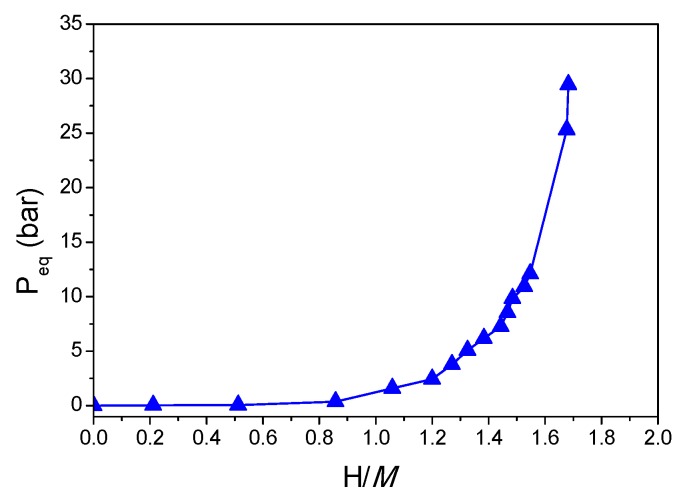
Pressure–composition isotherm for hydrogen absorption in Ti_0.325_V_0.275_Zr_0.125_Nb_0.275_ (BM) at 250 °C.

The Ti_0.325_V_0.275_Zr_0.125_Nb_0.275_ alloy synthesized by BM absorbs hydrogen forming a hydride phase with maximum capacity of 1.7 H/*M* (2.5 wt.%), as determined by PCI recorded at 250 °C up to 30 bar (Figure 3). The sample was exposed to air before loading; thus, high temperature was needed to increase the kinetics of reaction with hydrogen within reasonable time. This alloy absorbs hydrogen forming a hydride phase within a single-step transformation, similarly to the phase transition observed earlier for TiVZrNbHf [12].

**Figure 4:**
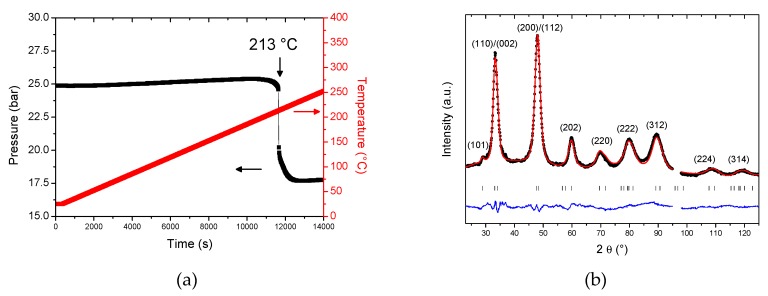
Pressure and temperature variations during deuterium absorption in Ti_0.325_V_0.275_Zr_0.125_Nb_0.275_ (BM) (the temperature ramp is 1 °C/min) (**a**) and the neutron powder diffraction pattern of the deuteride phase Ti_0.325_V_0.275_Zr_0.125_Nb_0.275_D_1.73_ (pattern recorded at room temperature) together with the Rietveld refinement (calculated pattern in red and the difference curve in blue) (**b**). The excluded zone from 95 to 97.5° is due to the powder diffraction contribution from the sample environment.

In order to analyze the crystalline structure of the hydride/deuteride phase, we have performed neutron powder diffraction at D1B (ILL). We have exposed the initial BM alloy to 25 bar of deuterium while heating with a constant temperature ramp of 1 °C/min (Figure 4a). At around 200 °C the alloy starts to absorb deuterium within one step with a maximum rate around 213 °C and a deuterium capacity of 1.73 H/*M*, which is in good agreement with the PCI measurement at 250 °C. The neutron powder diffraction was carried out on the deuteride phase after cooling at room temperature (Figure 4b). We have also recorded the neutron diffraction pattern for the initial alloy; however, the quality of the diagrams was very poor in terms of intensity/background ratio. This is not surprising since the TiVZrNb equimolar composition was proposed as low neutron scattering cross section material for in-core structural components for nuclear reactors over 1 million equimolar combinations [16]. More precisely, this alloy possesses a high transparency to thermal neutron which explains the poor quality of neutron powder diffraction. However, after deuterium absorption, the quality of the diffraction pattern is sufficient to perform Rietveld refinement (Figure 4b). All diffraction peaks of the deuteride phase could be indexed in a *bct* structure with the lattice parameters *a* = 3.167(1) and *c* = 4.371(1)Å (Table 1). The deuterium atoms occupy the tetragonal interstitial sites of the *bct* structure, in agreement with our previous study on TiVZrNbHf [12]. 

**Figure 5:**
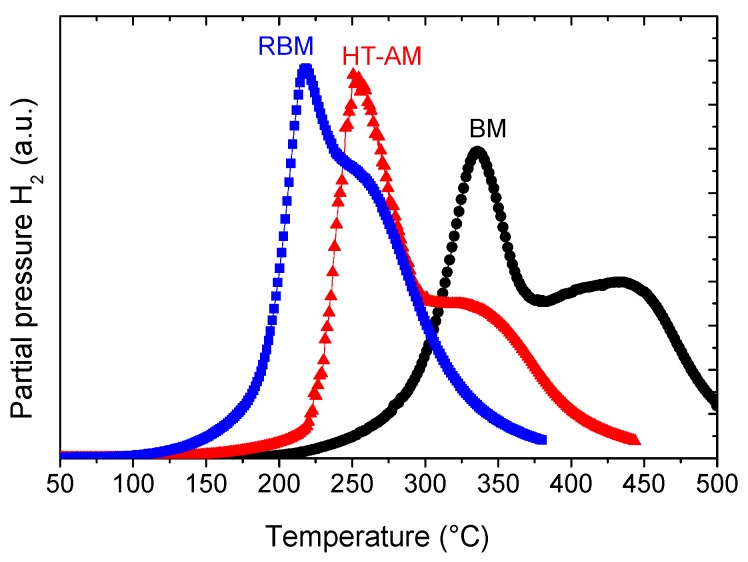
Thermo-desorption spectra of hydrogenated Ti_0.325_V_0.275_Zr_0.125_Nb_0.275_ alloys synthesized by BM (circles) and HT-AM (triangles) together with RBM hydride (squares) recorded with a temperature ramp of 2.6 °C/min.

Once fully hydrogenated, the hydrogen desorption properties were studied by the TDS technique. The spectrum recorded with a temperature ramp of 2.6 °C/min is displayed in Figure 5. The desorption profile shows two main peaks with a maximum desorption rate at around 330 °C and a second peak at 430 °C. The crystalline structure of the desorbed sample was analyzed by XRD and Rietveld refinement (Figure S4). The desorbed sample fully recovers the initial *bcc* structure with the lattice parameter *a* = 3.268(1)Å, close to the initial one (Table 1), proving complete reversibility of hydrogen absorption/desorption in this alloy. The phase transition during hydrogen absorption/desorption corresponds to *bcc* ↔ *bct*, in agreement with previous findings for refractory *bcc* high entropy alloys [7,12].

### 2.4. Hydrogen Sorption Properties of the TiVZrNb Alloy Prepared by HT-AM 

**Figure 6:**
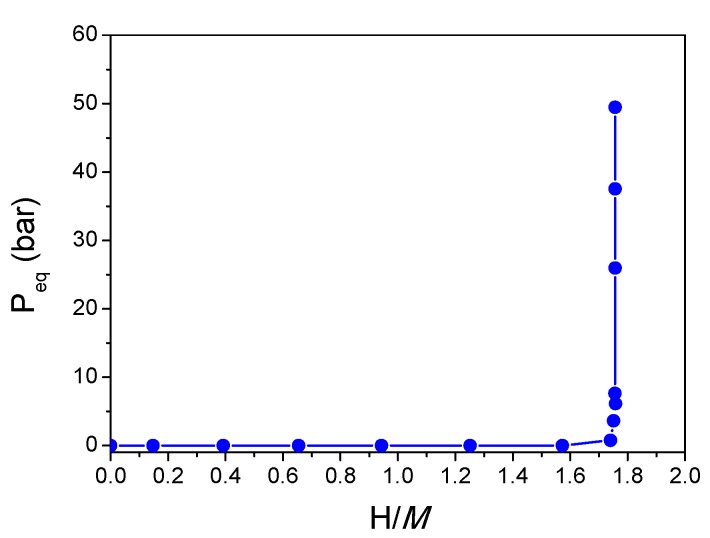
Pressure–composition isotherm for hydrogen absorption in Ti_0.325_V_0.275_Zr_0.125_Nb_0.275_ (HT-AM) at 25 °C.

The alloy obtained by HT-AM starts to absorb hydrogen at room temperature with relatively fast kinetics. The maximum uptake is around 1.75 H/*M* (2.5 wt.%) at room temperature, which is comparable to the capacity of the BM alloy, but this value was only reached at high temperature, 250 °C. The PCI curve recorded at 25 °C shows a single absorption plateau at low pressure (Figure 6). 

The crystalline structure after hydrogen absorption was determined by XRD (Figure S5). The hydride phase adopts a *bct* structure with the lattice parameters *a* = 3.168(1) and *c* = 4.523(1) (Table 1), in good agreement with the structure found by neutron powder diffraction on the hydrogenated BM alloy. 

The hydrogen desorption properties were tested by TDS, as shown in Figure 5. The two-peak desorption profile is very similar to the hydrogenated BM alloy but with faster kinetics since all peaks are shifted to lower temperatures. The maximum desorption rate is around 250 °C, which is 80 °C lower than hydrogenated BM alloy, and the second desorption peak occurs at 320 °C. The crystalline structure after desorption was determined by XRD (Figure S6) and the lattice parameters are listed in Table 1, as calculated from Rietveld analysis. The sample reversibly desorbs hydrogen and retrieves the initial *bcc* structure with *a* = 3.253(1) Å, very close to the value of pristine alloy (*a* = 3.261(1) Å). Moreover, the small impurity corresponding to the stars in Figure 1a completely disappears after hydrogen absorption/desorption. 

In summary, the HT-AM alloy has better absorption and desorption properties than the BM one: the absorption is faster, allowing the acquisition of a PCI curve at room temperature and the desorption occurs at lower temperature as compared to the BM hydride. Although both samples are exposed and manipulated in air, this behavior might be due to the larger surface area of BM alloy (powder), which is more prone to oxidation than the HT-AM material (very coarse powder). 

### 2.5. Hydrogen Sorption Properties of the TiVZrNbH_1.8_ Hydride Prepared by RBM

RBM allows one to directly prepare the hydride phase with a very good chemical homogeneity as proven by the EDX chemical mapping (Figure S7). The hydrogen desorption properties have been measured by TDS (Figure 5). This curve was measured after activation (one cycle of desorption/absorption) and shows similar profile to the other two materials but with the onset temperature at 185 °C. Both desorption peaks of RBM hydride occur at lower temperature than the previous materials: 220 and 260 °C. This proves that RBM method allows preparation of hydrides with better kinetics of desorption than the hydrogenated BM and HT-AM alloys. The desorbed material is a single-phase *bcc* structure, as analyzed by XRD and corresponding Rietveld refinement (Figure S8). The lattice parameter is *a* = 3.277(1) Å (Table 1), which is close to the values of the pristine alloy and the desorbed phases prepared by either BM or HT-AM methods.

**Figure 7:**
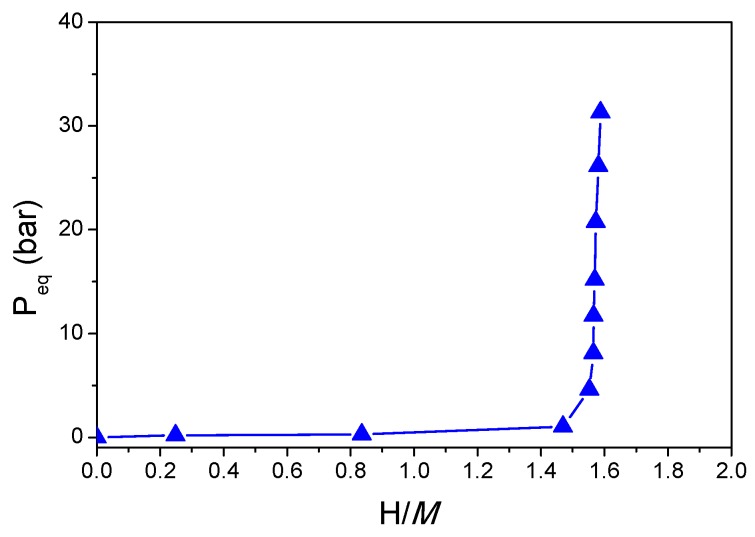
Pressure–composition isotherm for hydrogen absorption in Ti_0.325_V_0.275_Zr_0.125_Nb_0.275_ (RBM) at 25 °C.

The desorbed phase reversibly absorbs hydrogen at room temperature within one step, as demonstrated by the PCI curve in Figure 7. This behavior is similar to the previous alloys prepared by different synthetic methods proving that this MPEA composition shows a single step reaction with hydrogen from a *bcc* alloy to a *bct* hydride, irrespective of the preparation route.

**Figure 8:**
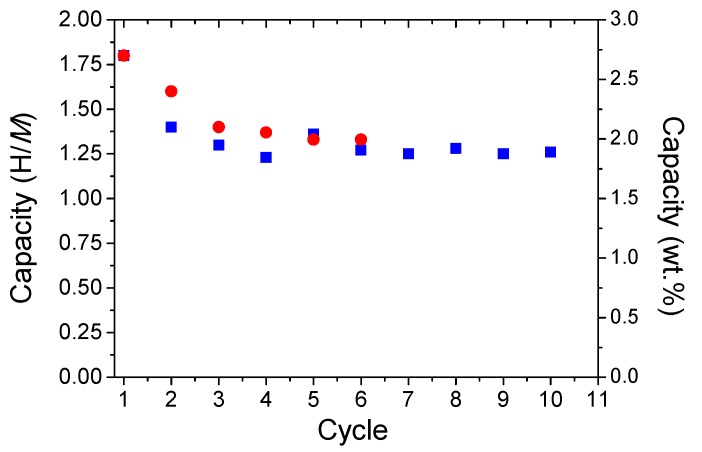
The evolution of the capacity during cycling under different conditions: absorption under 1 bar (circles) and 40 bar H_2_ pressure (squares) at room temperature and desorption under vacuum at 400 °C.

However, the maximum capacity is only 1.6 H/*M*, which is smaller than the initial uptake of 1.8 H/*M*, pointing out a fading of capacity during cycling. Thus, the cycling behavior was studied for this sample under different conditions: absorption under either 1 bar or 40 bar H_2_ pressure at room temperature and desorption under dynamic vacuum at 400 °C (Figure 8). Indeed, during the first three cycles, a constant decrease of the capacity from 1.8 H/*M* (2.7 wt.%) to around 1.25 H/*M* (2.0 wt.%) is noticed, followed by stabilization, irrespective of the absorption conditions. This proves that the reversibility of the reaction can be maintained at relatively good value even under atmospheric absorption conditions (1 bar H_2_ and 25 °C). The chemical homogeneity of the material after ten cycles was verified by EDX mapping and no phase segregation could be noticed (Figure S9).

Since the RBM material can reversibly absorb hydrogen, we have determined the activation energy of desorption *E_a_* via the Kissinger method [18]. The recorded TDS spectra and corresponding Kissinger plot are shown in Figure S10. From the slope of the linear fit, a value of 153 (±10) kJ/mol was found for *E_a_*.

### 2.6. SQS-DFT Modeling

The hydride formation in the equimolar TiVZrNb has been studied by coupling several methodologies, including the special quasirandom structure (SQS) and the density functional theory (DFT). First of all, an investigation of the most representative SQS cells has been done. Taking into account the *bcc* phase with four elements in equimolar composition, cluster expansion formalism was used to estimate the validity of the SQS generation as a function of the number of atoms by unit-cell. Considering about 42 different clusters, in which there are 24 different first neighbor pairs, each SQS-cell merit was estimated with the root means square (RMS) of the 42 correlation functions in comparison with a theoretical random distribution. It is shown that a number of 100 atoms of the representative host structure is sufficient to be under an RMS of 0.1, leading to a full cell of 300 atoms for the hydride phase with H/*M* = 2. Using the generated SQS-cell without hydrogen, the DFT calculation of the random TiVZrNb solid solution in *bcc* structure was performed using pseudopotential method (see details in Section 4). Volume and internal positions were allowed to be relaxed, keeping the original host structure symmetry. From the difference of total energies with the pure elements in their stable reference state, the calculation of the heat of formation was obtained, Δ_f_*H*(TiVZrNb)^bcc^ = +11.0 kJ/mol.

The ordered dihydrides were investigated by adding hydrogen in all tetragonal interstitials of the *bct* and *fcc* structures, with ThH_2_ and CaF_2_ prototypes, respectively. From the DFT calculations, the heats of formation are equivalent whatever the prototype with a quite exothermic reaction of Δ_f_*H*(TiVZrNbH_2_) = −36.9 kJ/mol-H. DFT accuracy does not allow one to distinguish the most stable phase among ThH_2_ and CaF_2_ structures since their difference of energy is about 0.1 kJ/mol. The relaxed cell parameters for all calculated structures (initial alloy—*bcc*, hydride phase—*bct* or *fcc*) are in good agreement with the experimental ones (see Table 1).

## 3. Discussion

Two MPEAs with the composition Ti_0.325_V_0.275_Zr_0.125_Nb_0.275_ were prepared by HT-AM and BM under Ar for 60 min and they possess a *bcc* structure with very close lattice parameter (Table 1). The slight difference might be explained by the small impurity present in the HT-AM alloy (marked with stars in Figure 1a), whereas the BM sample is single-phase. The impurity in the HT-AM alloy seems to have the same *bcc* structure as the main phase but with slightly different chemical composition and smaller lattice parameter since the two main diffraction peaks, (110) and (211), have small shoulders at higher 2θ angles. Interestingly, this impurity completely disappears after exposure to high temperature (typically 400 °C for hydrogen desorption) and the lattice parameter shrinks from 3.261 to 3.253 Å possibly due to improved chemical homogeneity. The lattice parameters of the alloys obtained experimentally (HT-AM and BM) are slightly smaller than the value provided by SQS-DFT modeling (Table 1). This small dissimilarity may be accounted for by different chemical composition between the prepared alloys and the equimolar formula used for modeling. The real alloys contain less Zr, which is the largest element among all components, than the equimolar composition. Thus, a decrease of the lattice parameter for the real alloy is expected.

Another interesting feature is that the broadening of the peaks of BM alloy are only slightly larger than the HT-AM one (Figure 1a,b). This is surprising since the BM method is well known to produce materials with crystallite sizes in the nanometer range and consequently rather large diffraction peaks, whereas the HT-AM alloys are crystalline materials with large crystallite sizes and sharp peaks. Along with the instrumental XRD contribution, the peak broadening of MPEA materials can be explained by the chemical disorder introduced by the random distribution of several elements on the same crystallographic site, the large lattice strain due to the mixing of many metals with different atomic radii and the small crystallite sizes. We hypothesize that the slightly larger width of the peaks from BM alloy may be explained by large lattice strain and/or small crystallites as a consequence of the ball milling process.

The calculated heat of formation of equimolar solid solution was found Δ_f_*H*(TiVZrNb)^bcc^ = +11.0 kJ/mol. The equivalent heat of mixing in the *bcc* phase is Δ_mix_*H*(TiVZrNb)^bcc^ = +6.5 kJ/mol, quite close to the value of King et al. at +4.0 kJ/mol obtained by a similar approach [16]. The positive value of Δ_f_*H* indicates that the phase is not stable at 0 K and needs an entropy contribution to be stabilized. In fact, if we consider that the *bcc* phase is stable if Δ*G =* Δ_f_*H* − *T*Δ*S* < 0 and that the entropy is only described by the configuration Boltzmann formula: $\Delta S=\;-R\;\overset{N}{\underset{i=1}{\sum }}x_{i}\ln x_{i}$, with $x_{i}=\frac{1}{N}$ and N=4, then an equivalent temperature of about 1000 K is necessary to reach Δ*G* < 0. Similar positive formation energy was calculated for other HEA systems; for example, the classical Cantor Alloys with Δ_f_*H*(CoCrFeMnNi)^FCC^ = +7.5 kJ/mol [14]. This finding may explain that the present alloy obtained by HT-AM is stable under our synthetic conditions (high temperature melting followed by rapid quench to room temperature). However, this conclusion cannot be extrapolated to the alloy prepared by BM which is a well-known method for the synthesis of materials far for thermodynamic equilibrium.

All studied alloys absorb hydrogen forming a hydride phase (maximum 1.75 H/*M*) within a single step transformation, as clearly demonstrated by the PCI measurements. This is in contrast with classical *bcc* alloys that absorb hydrogen, forming a monohydride (H/*M* = 1) at very low pressure and then a dihydride (H/*M* = 2) at higher pressure [19,20]. This behavior is similar to the phase transition observed earlier for TiVZrNbHf [7,12], although the maximum capacity is reduced. The latter composition can absorb hydrogen forming a *bct* hydride phase with maximum 2.5 H/*M* [7].

Due to the low neutron scattering cross section of all four elements, X-rays diffraction data provide a more reliable assessment of long range order of the metal lattice, whereas the neutron diffraction data gives consistent information about the long range structure of deuterium atoms occupying the interstitial sites of the metal lattice. We proposed that the hydride phase is *bct* and the deuterium atoms occupy the tetrahedral interstitial sites. However, it was recently claimed in the literature that other refractory MPEAs form *fcc* hydrides [8,10,13,21]. This is not surprising since our XRD analyses can be performed based on both *fcc* and *bct* lattices and the *bct* can be seen as a slightly distorted *fcc* structure. However, the use of the *bct* lattice gave better refinement results than the *fcc* one. Thus, we conclude that the phase transition during the single step hydrogen absorption/desorption in the Ti_0.325_V_0.275_Zr_0.125_Nb_0.275_ MPEA corresponds to *bcc* ↔ *bct*, which is a new reaction pathway in the metal–hydrogen system.

Moreover, the single-phase *bct* hydride phase can be directly prepared by RBM under high hydrogen pressure within only 60 min of process. The desorption properties of this phase are better than the hydrides of BM and HT-AM alloys, as proven by TDS (Figure 5). The maximum desorption temperature is 213 °C (heating rate 2.6 °C/min) and the activation energy of desorption is 153 (±10) kJ/mol, in good agreement with the value reported recently for the equimolar TiVZrNb alloy [13]. Furthermore, these results prove that the TDS desorption profile measured for the same hydride composition strongly depends on the synthetic method. Consequently, many factors, such as the microstructure, the surface passivation, and the sample history, influence the maximum desorption rate. In this context, it is worth comparing our results with the hydrogen desorption properties from the hydride of equimolar TiVZrNb recently reported by Nygård et al. [10]. The hydride of the equimolar composition shows a maximum desorption around 275 °C for a heating rate of 10 °C/min. This maximum temperature must be compared to desorption results recorded with same or close temperature ramp. In our case, the nonequimolar RBM hydride has a maximum desorption rate at 230 °C for a heating ramp of 11.6 °C/min (Figure S10). The faster desorption properties for our hydride might be a consequence of both decreasing the Zr content in the chemical composition and microstructure refinement by the ball milling process.

The absorption/desorption cycling of the RBM hydride shows promising properties at modest hydrogen absorption conditions (1 bar at room temperature). Despite the 26% fading of the capacity during the first cycles, this materials has a stable reversible uptake around 2 wt.% for further cycling, which is comparable to TiFeH_2_ hydride [22]. Moreover, the EDX chemical mapping has proven no phase segregation after ten cycles. All these features make the *bcc* MPEAs based on refractory elements and related hydrides very promising solid-state stores with capability to rapidly absorb hydrogen at room temperature.

In conclusion, we have shown that the mechanical alloying under reactive atmosphere is an appropriate synthetic tool for the preparation of hydrides of MPEAs starting from pure elemental powders, as also reported earlier by Zepon et al. for MgZrTiFe_0.5_Co_0.5_Ni_0.5_ [21]. Due to the versatile nature of this technique together with the fast and easy implementation, these results open the route to the study and discovery of a tremendous number of hydrides of MPEAs with a wide range of chemical compositions, large lattice distortions, and improved hydrogen storage performances.

## 4. Materials and Methods

The Ti_0.325_V_0.275_Zr_0.125_Nb_0.275_ alloy was synthesized by three different methods: high-temperature arc-melting under inert gas (HT-AM), high energy ball milling under Ar (BM), and reactive ball milling under H_2_ atmosphere (RBM).

The HT-AM alloy was prepared from stochiometric bulk pieces of the constituent elements: Ti (Kurt J. Lesker, 99.995%), V (ChemPur, 99.9%), Zr (ChemPur, 99.8%), and Nb (Alfa Aesar, 99.95%) in an Ar atrmosphere. Prior to melting the sample, a Ti-getter piece was melted to reduce O_2_ content in the chamber. The sample was then remelted five times and flipped between each melting step to improve the homogeneous distribution of the elements. The alloy was further manipulated in air.

The BM alloy was prepared by weighing 5 g of total mass starting from pure elemental powders provided by Alfa Aesar: Titanium (99.9%, <100 μm), Vanadium (99.7%, 1–3 mm), zirconium (99.5%, <150 μm), and Niobium (99.8%, <250 μm). The metal powders were loaded inside a glove box under Ar without air exposure. Hardened stainless steel vial and 7 mm stainless steel balls in a weight ball-to-powder ratio 26:1 have been used. The powder mixture was mechanically alloyed using a Fritsch Pulverisette 7 planetary milling instrument running at 700 rpm. The milling was conducted in repeated cycles of 15 min for a total of 1 h. At each step of ball milling, the powder was hand-scraped from the inner-wall of the vial to ensure homogeneity of the alloy. After synthesis, the BM sample was manipulated in air.

The synthesis of RBM samples was carried out in a high-pressure milling vial equipped with gas pressure and temperature sensors commercialized by Evicomagnetics [17,23] and 12 mm stainless steel balls with a ball-to-powder ratio of 60:1. The vial was loaded up to 70 bar of H_2_ (6 N) and the alloying was carried out in 2 h of continuous milling in a Fritsch Pulverisette 4 planetary milling at a disk and vial rotation speed of 400 rpm and −2 rpm (800 rpm relative to disk), respectively. The initial powder metals were loaded under protective atmosphere inside an Ar glove box and the final product was further manipulated and stored in the glove box due to the high pyrophoricity of this material.

The hydrogen storage capacities were measured by pressure–composition isotherms (PCI) using an automated Sievert apparatus PCTPro-2000. The sample holder is a home-made stainless steel container closed with a metal seal and the temperature controlled by a resistance furnace. The HT-AM alloy in the form of very coarse powder was activated by heating under dynamic vacuum at 340 °C for 2 h followed by two absorption/desorption cycles (absorption at 50 bar H_2_ pressure and 25 °C; desorption under dynamic vacuum at 400 °C for at least 3 h).

The hydrogen desorption properties have been studied by thermal desorption spectroscopy (TDS) using a homemade instrument, as described in a previous report [24]. Ten milligrams of sample were loaded in an aluminum tube inside a glove box and then mounted on the instrument without air exposure. Secondary vacuum was employed to pump out the system down to 10^−6^ mbar base pressure. The temperature was controlled by a resistance furnace and heated up to 450 °C with constant heating rates from 1 to 12 °C/min. The partial pressure of H_2_ was recorded by a mass spectrometer as a function of temperature.

The crystalline structure of samples was studied by powder X-ray diffraction (XRD) using a D8 advance Bruker diffractometer (Cu K_α_ radiation, Bragg-Brentano Geometry).

Powder neutron diffraction experiments were performed on the neutron beamline D1B at the Institut Laue-Langevin, Grenoble (France) with λ = 1.28 Å and 0.1° step in 2θ, from 1° < 2θ < 128°. The stainless steel cell containing the sample is coupled to a homemade volumetric apparatus (Sievert’s device) to determine the deuterium uptake. The alloy was submitted to 25 bar of deuterium (D_2_) at a constant heating rate of 1 °C/min up to 350 °C. Due to the large scattering contribution from the stainless steel cell, only ex situ measurements of the sample after deuteration were performed using a vanadium container. The crystalline structure of the alloys/hydrides/deuterides were determined by Rietveld analysis using the software Fullprof on both X-ray and neutron diffraction patterns [25].

The microstructure was analyzed by scanning electron microscopy (SEM) using a Zeiss Merlin microscope complemented by energy dispersive spectroscopy (EDS) from Oxford Instruments. Powder samples were immobilized in epoxy resin and coated with 2 nm of Pd. A chemical mapping was performed on the samples before and after cycling to determine the chemical homogeneity.

The SQS [26] cell for the equimolar TiVZrNb alloy was generated using the MCSQS tools of the ATAT package [27]. We choose the equimolar composition, which is slightly different from the real alloy’s concentration, in order to minimize the number of atoms of the representative host structure. The DFT calculations have been performed using the projected augmented wave method included in the VASP code [28,29] within the generalized gradient approximations (GGA). The Perdew–Burke–Ernzerhof (PBE) functional [30] has been chosen for the exchange and correlation energy functional. An energy cutoff of 600 eV was used for the plane wave basis set involving a dense grid of k-points in the irreducible wedge of the Brillouin zone.

## Figures and Tables

**Table 1:** Synthesis details and structural data for Ti_0.325_V_0.275_Zr_0.125_Nb_0.275_ alloy and related hydride and desorbed phases as prepared by high temperature arc melting (HT-AM), ball milling under Ar (BM), and reactive ball milling under H_2_ (RBM). The lattice parameters of the initial and hydride phases calculated by SQS-DFT are also given.

Sample	Preparation Method/Modeling	Phase	Structure	Space Group	Lattice Parameters (Å)
Ti_0.325_V_0.275_Zr_0.125_Nb_0.275_	HT-AM	alloy	*bcc*	*Im-3m*	*a* = 3.261(1)
Ti_0.325_V_0.275_Zr_0.125_Nb_0.275_H_1.75_		hydride	*bct*	*I4/mmm*	*a* = 3.168(1) *c* = 4.523(1)
Ti_0.325_V_0.275_Zr_0.125_Nb_0.275_		desorbed	*bcc*	*Im-3m*	*a* = 3.253(1)
Ti_0.325_V_0.275_Zr_0.125_Nb_0.275_	BM	alloy	*bcc*	*Im-3m*	*a* = 3.270(1)
Ti_0.325_V_0.275_Zr_0.125_Nb_0.275_H_1.7_		hydride	*bct*	*I4/mmm*	*a* = 3.167(1) *c* = 4.371(1)
Ti_0.325_V_0.275_Zr_0.125_Nb_0.275_		desorbed	*bcc*	*Im-3m*	*a* = 3.268(1)
Ti_0.325_V_0.275_Zr_0.125_Nb_0.275_H_1.8_	RBM	hydride	*bct*	*I4/mmm*	*a* = 3.194(1) *c* = 4.448(1)
Ti_0.325_V_0.275_Zr_0.125_Nb_0.275_		desorbed	*bcc*	*Im-3m*	*a* = 3.277(1)
TiVZrNb	SQS-DFT	alloy	*bcc*	*Im-3m*	*a* = 3.29
TiVZrNbH_2_		hydride	*bct*	*I4/mmm*	*a* = 3.19 *c* = 4.52
TiVZrNbH_2_		hydride	*fcc*	*Fm-3m*	*a* = 4.51

## Supplementary Materials

The following are available online. Figure S1. The evolution of XRD patterns of equimolar TiVZrNb alloy after 15, 30, 45, 60, 120, and 150 min of ball milling under Ar; Figure S2. The hydrogen uptake profile during reactive ball milling under H_2_ atmosphere with 400 rpm (a) and the XRD pattern after 120 min of milling process (b); Figure S3. The XRD pattern of the RBM hydride sample and the corresponding Rietveld refinements using fcc (a) and bct (b) structures; Figure S4. The XRD pattern of the hydrogenated BM sample after desorption by TDS and the corresponding Rietveld refinement; Figure S5. The XRD pattern of the hydrogenated HT-AM sample and the corresponding Rietveld refinement; Figure S6. The XRD pattern of the hydrogenated HT-AM sample after desorption by TDS and the corresponding Rietveld refinement; Figure S7. The EDX chemical mapping of the hydride obtained by RBM; Figure S8. The XRD pattern of the RBM hydride after desorption by TDS and the corresponding Rietveld refinement; Figure S9. The EDX chemical mapping of the material obtained by RBM after 10 hydrogen desorption/absorption cycles; Figure S10. TDS spectra of hydrogen desorption from RBM material recorded with several heating rates (left) and related Kissinger plot (right).
