# Supplementary material for: TiVZrNb Multi-Principal-Element Alloy: Synthesis Optimization, Structural, and Hydrogen Sorption Properties
Source: Molecules. 2019 Jul 31;24(15):2799. doi: 10.3390/molecules24152799 (PMC6695880; doi:10.3390/molecules24152799)
Supplement: Supplementary file 1 [file molecules-24-02799-s001.pdf]

Supplementary Materials

TiVZrNb multi-principal-element alloy: synthesis, structural and hydrogen sorption properties

Jorge Montero[1], Claudia Zlotea[1,*], Gustav Ek[2], Jean-Claude Crivello[1], Lætitia Laversenne[3], Martin Sahlberg[2]

[1] Université Paris Est, ICMPE (UMR 7182), CNRS, UPEC, F- 94320 Thiais, France; montero@icmpe.cnrs.fr, zlotea@icmpe.cnrs.fr

[2] Department of Chemistry, Uppsala University, Box 523, SE-75120 Uppsala, Sweden; martin.sahlberg@kemi.uu.se, gustav.ek@kemi.uu.se

[3] Univ. Grenoble Alpes, CNRS, Grenoble INP, Institut Néel, 38000 Grenoble, France; laetitia.laversenne@neel.cnrs.fr

* Correspondence: zlotea@icmpe.cnrs.fr

Figure 1. The evolution of XRD patterns of equimolar TiVZrNb alloy after 15, 30, 45, 60, 120 and 150 minutes of ball milling under Ar.

Figure 2. The hydrogen uptake profile during reactive ball milling under H_2 atmosphere with 400 rpm (a) and the XRD pattern after 120 minutes of milling process (b).

Figure 3. The XRD pattern of the RBM hydride sample and the corresponding Rietveld refinements using *fcc* (a) and *bct* (b) structures.

Figure 4. The XRD pattern of the hydrogenated BM sample after desorption by TDS and the corresponding Rietveld refinement.

Figure 5. The XRD pattern of the hydrogenated HT-AM sample and the corresponding Rietveld refinement.

Figure 6. The XRD pattern of the hydrogenated HT-AM sample after desorption by TDS and the corresponding Rietveld refinement.

Figure 7. The EDX chemical mapping of the hydride obtained by RBM.

Figure 8. The XRD pattern of the RBM hydride after desorption by TDS and the corresponding Rietveld refinement.

Figure 9. The EDX chemical mapping of the material obtained by RBM after 10 hydrogen desorption/absorption cycles.

Figure 10. TDS spectra of hydrogen desorption from RBM material recorded with several heating rates (left) and related Kissinger plot (right).

Figure 1. The evolution of XRD patterns of TiVZrNb alloy (equimolar composition) after 15, 30, 45, 60, 120 and 150 minutes of ball milling under Ar.

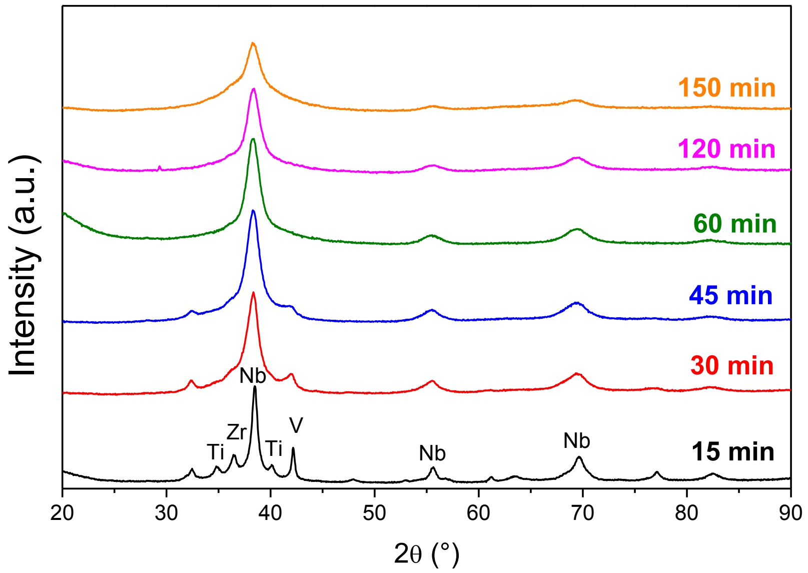

The elemental contributions present initially decrease steadily with time in favor of a main *bcc* phase. After 2h of ball milling the phase became partially amorphous.

Figure 2. The hydrogen uptake profile during reactive ball milling under H_2 atmosphere with 400 rpm (a) and the XRD pattern after 120 minutes of milling process (b).

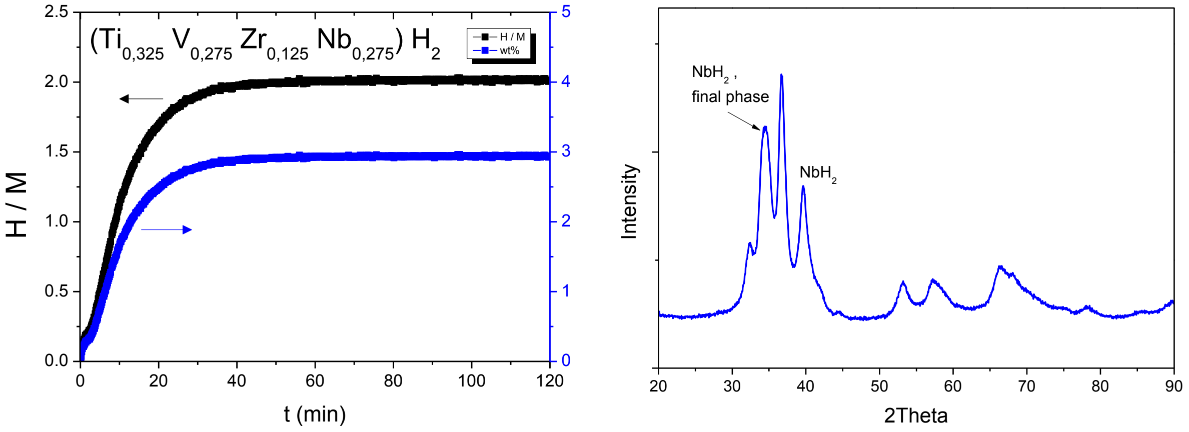

(a) (b)

Figure 3. The XRD pattern of the RBM hydride sample and the corresponding Rietveld refinements using *fcc* (a) and *bct* (b) structures. The goodness-of-fit χ^2 and R_{wp} factor are given in inset.

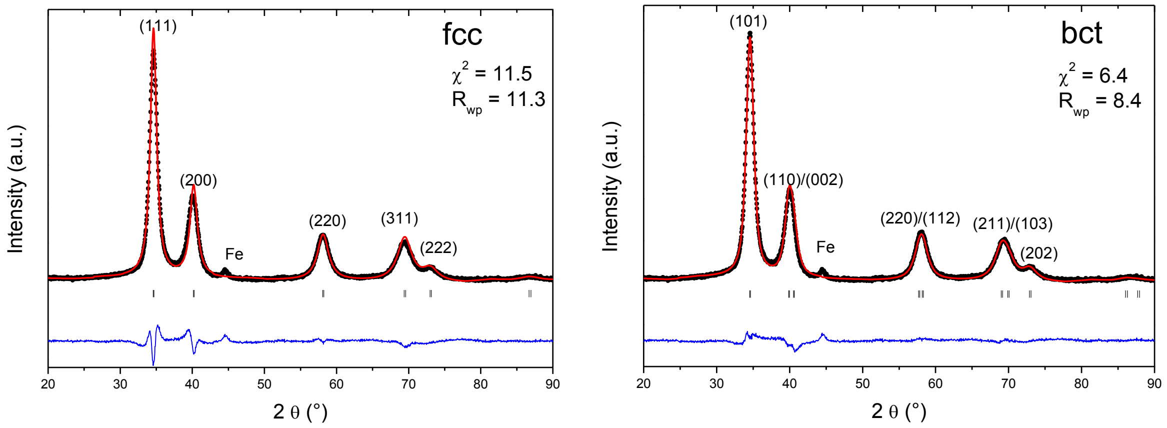

(a) (b)

Overall all diffraction peaks from the RBM sample can be indexed in either *fcc* (a_{fcc} = 4.496±1 Å) or *bct* lattice (a_{bct} = 3.194±1 Å, c_{bct} = 4.448±1 Å), and the latter structure can be understood as a slight distortion of the first one. The second peak at around 40 ° corresponds to two distinct peaks of the *bct* lattice (110 and 002) and consequently, can be used as the footprint of the *bct* distortion. Therefore, the close assessment of this peak might help distinguishing between the two lattices. A zoom of the two structural fits of this peak is given below and clearly indicates that the fit using the *bct* lattice better describes this peak whereas, the *fcc* one shows a shift of position and a too small FWHM.

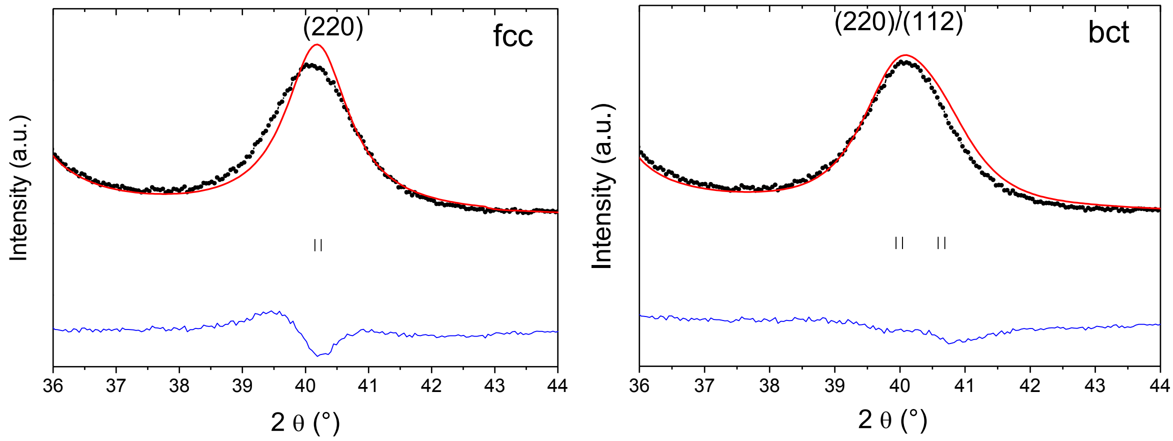

Moreover, the Rietveld refinements give better confidence factors for the *bct* lattice as compared to the *fcc*. In summary, the *bct* lattice is preferred and the tetragonal distortion is estimated as $(a_{bct} * \sqrt{2} - a_{fcc})/a_{fcc})$ along the *a* axis (~5%) and as $(c_{bct} - c_{fcc})/c_{fcc})$ along the *c* axis (~ -10%).

Figure 4. The XRD pattern of the hydrogenated BM sample after desorption by TDS and the corresponding Rietveld refinement with the *bcc* lattice. The goodness-of-fit χ^2 and R_{wp} factor are given in inset.

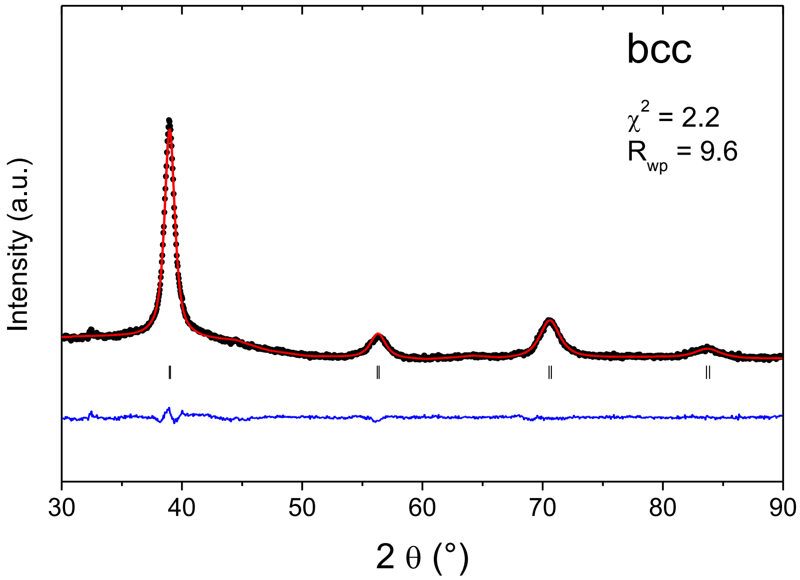

Figure 5. The XRD pattern of the hydrogenated HT-AM sample (after PCI at 25 °C) and the corresponding Rietveld refinement.

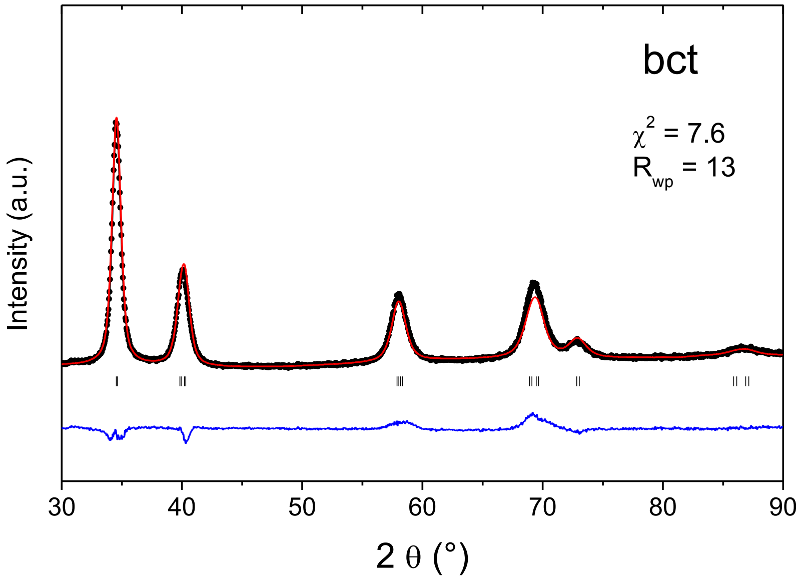

Figure 6. The XRD pattern of the hydrogenated HT-AM sample after desorption by TDS and the corresponding Rietveld refinement.

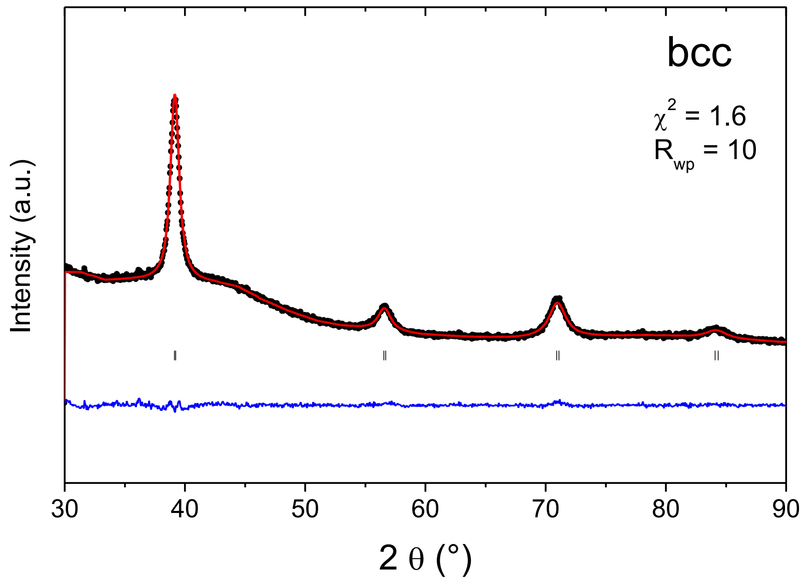

Figure 7. The EDX chemical mapping of the hydride obtained by RBM. Very good homogeneity among the four elements is noticed.

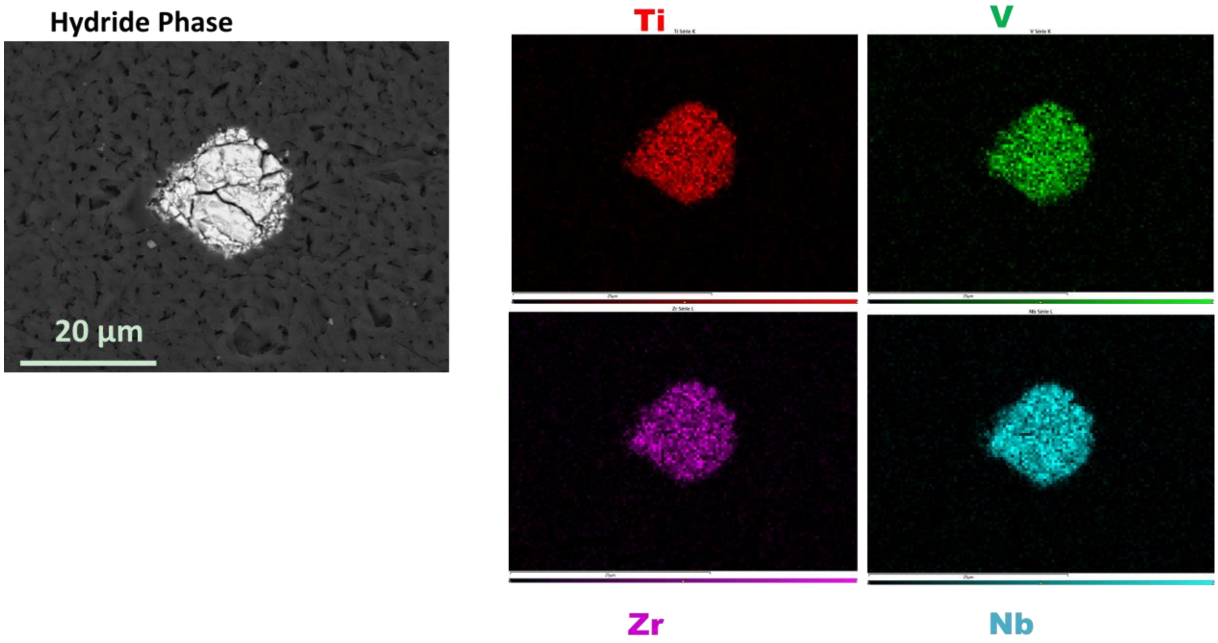

Figure 8. The XRD pattern of the RBM hydride after desorption by TDS and the corresponding Rietveld refinement.

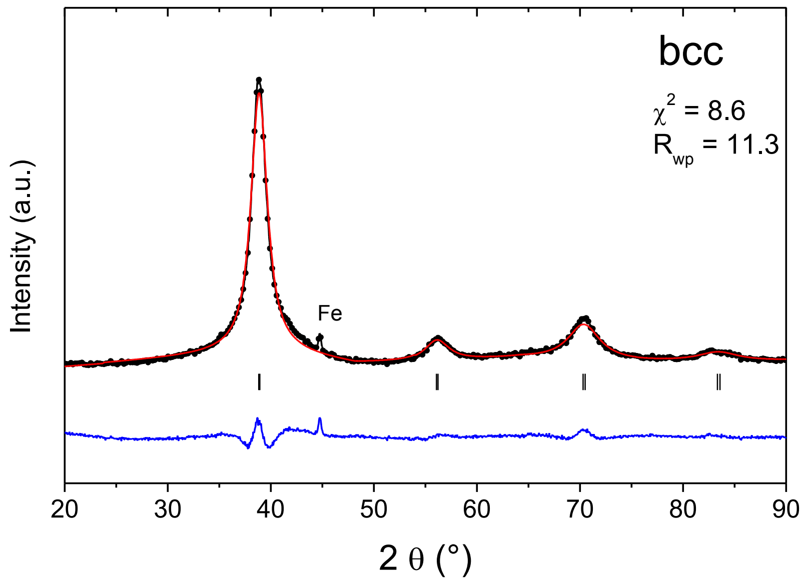

Figure 9. The EDX chemical mapping of the material obtained by RBM after 10 hydrogen desorption/absorption cycles. Very good homogeneity among the four elements and no phase segregation were observed.

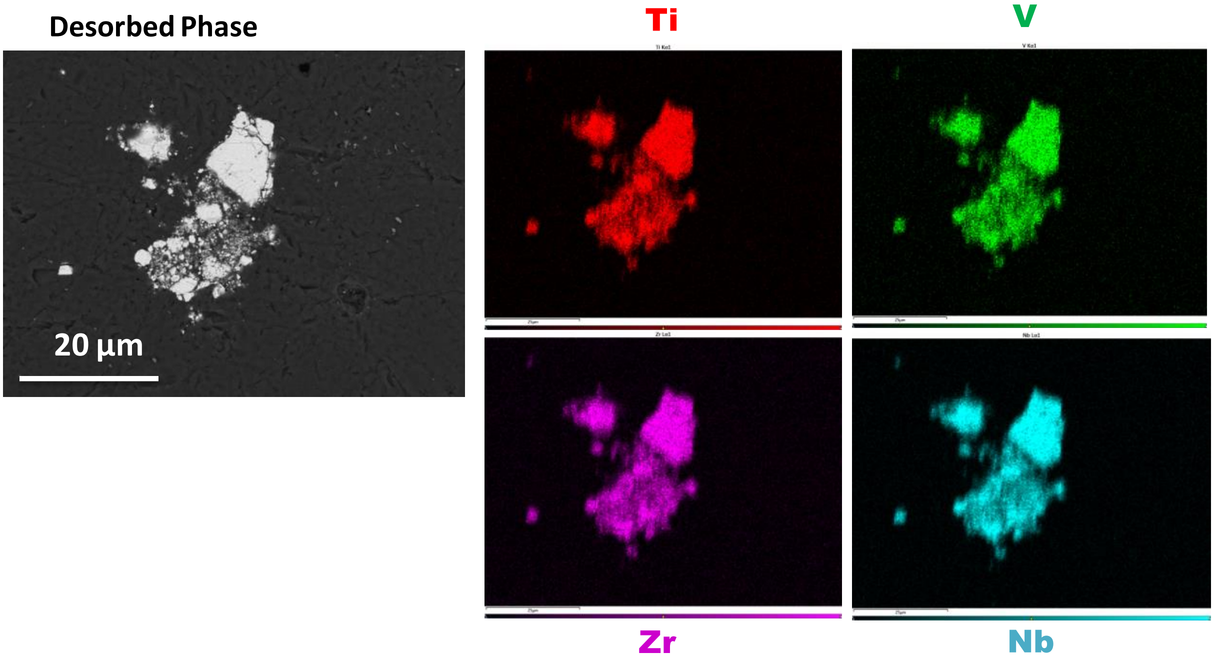

Figure 10. TDS spectra of hydrogen desorption from RBM material recorded with several heating rates (left) and related Kissinger plot (right).

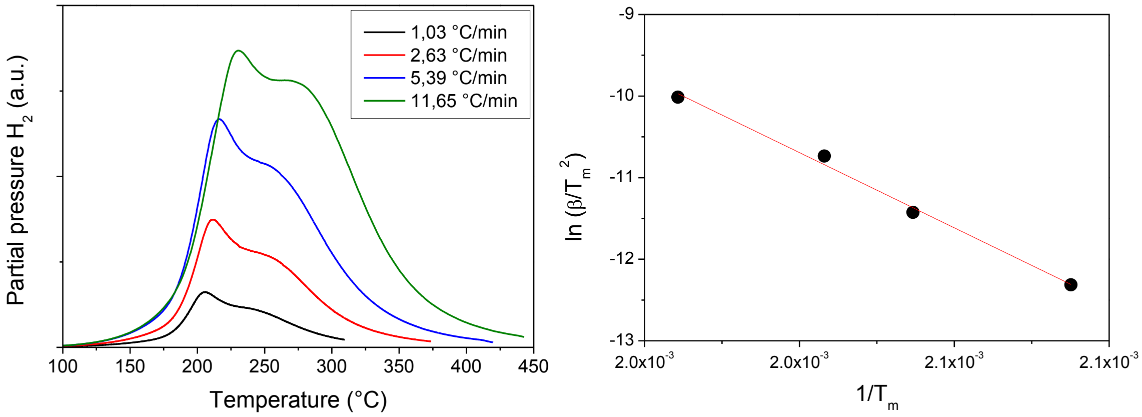

The Kissinger method [1] utilizes the characteristic temperature T_m at which the desorption rate reaches its maximum (the main desorption peak) at different linear heating rates, β, and it's used to determine the activation energy of desorption from the equation:

$$\ln(\beta / T_m^2) = -\frac{E_a}{RT} + \ln(k_0)$$

where E_a is the activation energy of desorption and k_0 is a reaction constant. Therefore, the logarithmic plot of β/T_m^2 vs. $1/T_m$ gives a straight line with a slope equal to E_a/R.

The activation energy of desorption from RBM hydride is 153 (±10) kJ/mol.

[1] R.L. Blaine, H.E. Kissinger, Homer Kissinger and the Kissinger equation, Thermochim. Acta. 540 (2012) 1–6. doi:10.1016/j.tca.2012.04.008.
